# Acute Kidney Injury Complicating Wasp Stings: A Report of Two Cases and Literature Review

**DOI:** 10.7759/cureus.37343

**Published:** 2023-04-09

**Authors:** Abdul Rehman Arshad, Amir Rashid

**Affiliations:** 1 Nephrology, Pak Emirates Military Hospital, Rawalpindi, PAK; 2 Internal Medicine, Pak Emirates Military Hospital, Rawalpindi, PAK

**Keywords:** wasp sting, rhabdomyolysis, pigment nephropathy, haemodialysis, envenomation

## Abstract

Acute kidney injury could occasionally complicate wasp stings. We describe two such cases. The first one developed acute kidney injury as a result of rhabdomyolysis and hemolysis, whereas the other patient developed acute kidney injury as part of multiorgan dysfunction syndrome resulting from shock and rhabdomyolysis. Both remained dependent on intermittent hemodialysis for a short period of time before recovering spontaneously. These cases highlight different pathophysiological processes leading to acute kidney injury, and the importance of timely diagnosis to achieve favourable clinical outcomes.

## Introduction

Wasps are commonly found in temperate climates as is seen in Pakistan. A comprehensive account of different types of wasps commonly encountered in Pakistan has been presented by Rafi et al. [1]. These insects generally sting once they feel endangered. The resulting clinical effects have a wide spectrum ranging from as mild as localized skin reaction to severe enough as to cause multiorgan dysfunction and death. Local epidemiological data is not available. Though many cases with systemic complications have been reported in literature from around the world, data from Pakistan is probably underreported. There is only one case report from our country on PubMed, that too of acute kidney injury (AKI) complicating multiple wasp stings [2]. Here we present an account of two cases complicated by AKI that resolved with conservative management and hemodialysis. This is followed by a review of relevant literature.

## Case presentation

Case 1

**Figure 1:**
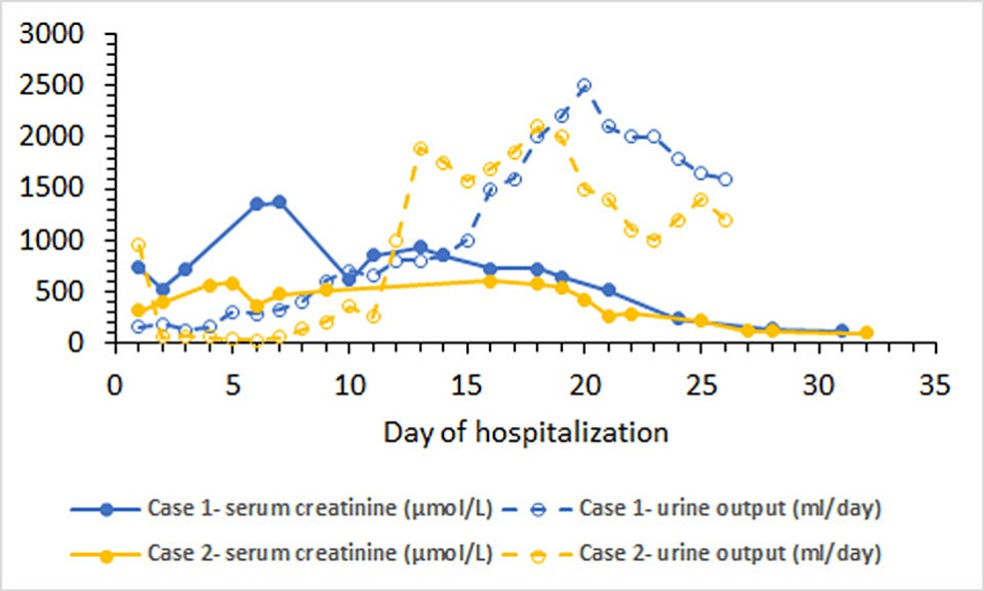
Trends in serum creatinine and daily urine output

A 23-year-old man was stung by wasps at multiple sites on his face, head and upper extremities while repairing a telephone cable outdoors. He was initially managed with intravenous antihistamines and hydrocortisone by a local doctor. Over the next 48 hours, he gradually developed nausea and vomiting and started passing reduced amounts of dark-coloured urine. He then reported to our hospital in the last week of October 2022. At that time, he was hemodynamically stable and had a blood pressure of 160/100 mmHg. There were multiple sting marks on his body. Systemic physical examination showed pedal edema and signs of pleural effusions bilaterally. Initial investigations revealed haemoglobin 15.2 g/dL (reference range: 13.5-16.5 g/dL), white cell count 22100/µL (reference range: 4000-11000/µL), platelets 349000/µL (reference range: 150000-400000/µL), serum urea 21 mmol/L (reference range: 2.1-7.1 mmol/L), creatinine 734 µmol/L (reference range: 53-115 µmol/L), sodium 133 mmol/L (reference range: 135-145 mmol/L), potassium 5.4 mmol/L (reference range: 3.5-5.1 mmol/L), creatinine kinase 25609 U/L (reference range: 25-192 U/L), lactate dehydrogenase 5332 IU/L (reference range:240-480 U/L), bilirubin 110 mmol/L (reference range: 2-17 mmol/L), alanine transaminase 53 U/L (reference range: up to 42 U/L), alkaline phosphatase 230 U/L (reference range: up to 150 U/L), total calcium 1.96 mmol/L (reference range: 2.2-2.6 mmol/L) and normal serum albumin and coagulation profiles. Arterial blood gases showed high anion gap metabolic acidosis. Urine dipstick examination and microscopy were normal. Ultrasound revealed structurally normal kidneys. Echocardiogram showed normal ejection fraction (60%). The patient was managed in a high-dependency unit. He had seven sessions of hemodialysis done via an internal jugular vein double-lumen catheter. His urine output improved gradually, followed by an improvement in renal functions. He did not develop any other complications during hospital stay and was discharged in the last week of November 2022. Trends in serum creatinine levels and daily urine output throughout the period of admission are shown in Figure 1. The patient was asymptomatic and had normal serum creatinine levels two months later.

Case 2

A 39-year-old man was attacked by multiple wasps on the upper part of his body while going to toilet in the countryside in October 2022. At the time of presentation to our hospital after approximately 16 hours, he was in shock with a blood pressure of 80/40 mmHg and a pulse of 130 beats per minute. There were signs of pleural effusions bilaterally and ascites. He was initially managed with intravenous normal saline, antihistamines and hydrocortisone. He was then admitted to the intensive care unit, where he was found to have evidence of multiorgan dysfunction. Investigations revealed haemoglobin 13.4 g/dL, white cell count 14500/µL, platelets 211000/µL, serum urea 15 mmol/L, creatinine 308 µmol/L, sodium 139 mmol/L, potassium 5.7 mmol/L, creatinine kinase 15870 U/L, lactate dehydrogenase 8140 U/L, bilirubin 60 mmol/L, alanine transaminase 3001 U/L, alkaline phosphatase 470 IU/L, calcium 2.02 mmol/L, international normalized ratio 2.1 and high anion gap metabolic acidosis. Urine dipstick examination showed proteins (+) and there were 8-10 red blood cells per high power field on microscopy. Ultrasound demonstrated structurally normal kidneys. Echocardiogram showed normal ejection fraction (60%). He was managed conservatively. However, over the next few days, the urine output remained low and renal functions continued to worsen progressively. Maximum serum creatinine level was 580 µmol/L, on the fifth day of admission. He had to be started on renal replacement therapy for fluid overload and required eight sessions of hemodialysis before renal functions started improving spontaneously. He was discharged from hospital on the 26th day and was doing well two months later, with normal renal excretory functions. Trends in serum creatinine levels and daily urine output throughout the period of admission are shown in Figure 1.

## Discussion

Wasp stings could occur accidentally or following an occupational exposure. A single sting generally causes allergic reactions/anaphylaxis, whereas increasing number of stings are associated with systemic complications. Mortality rates of up to 25% have previously been described in the latter group [3]. AKI after wasp stings is not that uncommon. Xie et al. have documented an incidence of 21% amongst a large cohort of admitted patients in China [4]. Multiple factors could contribute to renal injury, including hypotension, rhabdomyolysis, hemolysis or acute interstitial nephritis as a result of hypersensitivity reaction to the venom. Direct toxic effects of the venom should also be considered especially if serum creatinine kinase levels are normal. It is widely believed that several different toxin components such as phospholipases, hyaluronidases, polypeptides and amines are responsible for direct toxic effects as well as inciting an immuno-inflammatory cascade leading to these complications [5].

Clinical presentation is generally as oliguric AKI in 70% cases, with variable degrees of hematuria and pyuria [6]. The disease course is generally worse in the elderly and those with macroscopic hematuria, anaemia or shock [4,7]. Timely recognition and appropriate management are important to prevent the development of chronic kidney disease, which has been reported in one out of every 10 patients [8]. Yuan et al. described higher levels of alanine transaminase, aspartate transaminase, lactate dehydrogenase, creatinine phosphokinase (MB fraction), prothrombin time, and proteinuria on presentation to be predictive of this [9]. Once AKI has developed, treatment is generally supportive, with institution of renal replacement therapy as per standard indications. According to published literature, it is required in 55-91% of cases [10]. As with AKI secondary to other etiologies, no specific modality of renal replacement therapy has any specific advantage over the others. There is limited evidence to support the use of plasma exchange [11]. AKI should generally start improving within a week or two. It has been suggested that selected patients not responding to conservative management should have a kidney biopsy done to look for acute interstitial nephritis, since this generally requires corticosteroids and responds well to such treatment [12].

## Conclusions

Most of the wasp stings go unnoticed and are not reported because of generally benign consequences. However, patients with a greater sting burden are more likely to have systemic complications. AKI could be the only manifestation or could be a part of more widespread multiorgan dysfunction syndrome. Meticulous care is required to pick this up at an early stage. Many patients even require renal replacement therapy for a short period of time, but would eventually improve and regain normal renal excretory functions.

## References

[1] Rafi MA, Carpenter JM, Qasim M (2017). The vespid fauna of Pakistan. Zootaxa.

[2] Butt G, Ullah K, Kanwal K, Mudassir Mudassir (2014). Acute renal failure following multiple wasp stings. J Coll Physicians Surg Pak.

[3] Silva GB Jr, Vasconcelos AG Jr, Rocha AM (2017). Acute kidney injury complicating bee stings - a review. Rev Inst Med Trop Sao Paulo.

[4] Xie C, Xu S, Ding F (2013). Clinical features of severe wasp sting patients with dominantly toxic reaction: analysis of 1091 cases. PLoS One.

[5] Radhakrishnan H (2014). Acute kidney injury and rhabdomyolysis due to multiple wasp stings. Indian J Crit Care Med.

[6] Ruwanpathirana P, Priyankara D (2022). Clinical manifestations of wasp stings: a case report and a review of literature. Trop Med Health.

[7] Wang M, Prince S, Tang Y (2021). Macroscopic hematuria in wasp sting patients: a retrospective study. Ren Fail.

[8] Zhang L, Yang Y, Tang Y, Zhao Y, Cao Y, Su B, Fu P (2013). Recovery from AKI following multiple wasp stings: a case series. Clin J Am Soc Nephrol.

[9] Yuan H, Lu L, Gao Z, Hu F (2020). Risk factors of acute kidney injury induced by multiple wasp stings. Toxicon.

[10] Vikrant S, Parashar A (2017). Acute kidney injury due to multiple Hymenoptera stings-a clinicopathological study. Clin Kidney J.

[11] Dhanapriya J, Dineshkumar T, Sakthirajan R, Shankar P, Gopalakrishnan N, Balasubramaniyan T (2016). Wasp sting-induced acute kidney injury. Clin Kidney J.

[12] Kumar V, Nada R, Kumar S (2013). Acute kidney injury due to acute cortical necrosis following a single wasp sting. Ren Fail.

